# Determination of the Infectivity of Cryopreserved *Theileria annulata* Sporozoites in Tick Derived Stabilates Iran Ak-93 Strain, by In Vivo and In Vitro Methods

**Published:** 2019

**Authors:** Hossein MODIRROUSTA, Gholamreza HABIBI, Parviz SHAYAN, Asghar AFSHARI, Ali MIRJALILI, Mohamad ABDIGOUDARZI

**Affiliations:** 1. Department of Wild Life Research, Razi Vaccine and Serum Research Institute, Agriculture Research, Education and Extension Organization, Karaj, Iran; 2. Department of Parasite Vaccine Research and Production, Razi Vaccine and Serum Research Institute, Agriculture Research, Education and Extension Organization, Karaj, Iran; 3. Department of Parasitology, School of Veterinary, Tehran University, Tehran, Iran; 4. Department of Biotechnology, Razi Vaccine and Serum Research Institute, Agriculture Research, Education and Extension Organization, Karaj, Iran; 5. Department of Parasitology, Razi Vaccine and Serum Research Institute, Agriculture Research, Education and Extension Organization, Karaj, Iran

**Keywords:** *Theileria annulata*, *Hyalomma a. anatolicum*, Tick stabilate, RT-PCR

## Abstract

**Background:**

The protozoan parasite *Theileria annulata* is the causative agent of tropical theileriosis in cattle. Vaccination is recommended by administration of attenuated schizont-infected cell lines. The expected protective immunity post-vaccination can be demonstrated by challenge test through inoculation of highly virulent infective sporozoites. The aim of this study was to produce *Hyalomma anatolicum anatolicum* tick infected with *T. annulata* (local strain) for preparation of tick-derived sporozoite stabilates for molecular characterization and infectivity test assay.

**Methods:**

A local *T. annulata* strain was used for experimental infection of calves. A field isolate of *H. a. anatolicum* was isolated, laboratory-reared and infected by blood-feeding on *Theileria* infected above-mentioned calves. The infectivity of calf, tick and prepared stabilate were confirmed by clinical signs of theileriosis, microscopic inspection, RT-PCR and in vitro cell culture.

**Results:**

The tick stabilate was prepared and cryopreserved in liquid nitrogen. The infectivity of the tick stabilate was verified by in vivo bioassay, in vitro cell culture infection, microscopic inspection in salivary glands and RT-PCR assay. The in vitro produced cell line in this study was characterized by *T. annulata* Cytochrome b gene analyzing.

**Conclusion:**

The infectivity of a new prepared tick-derived sporozoite stabilate was confirmed in susceptible calves; by microscopically, post mortem, tick microscopic and molecular assays. Moreover, naïve PBMCs were transformed and proliferated by *T. annulata* infected tick stabilate to immortal *T. annulata* schizont infected cell line. The potent infective sporozoite tick derived stabilate could be used for vaccine efficacy and challenge test as well as in vaccine development.

## Introduction

T*. annulata* is an obligate intracellular apicomplexan protozoan parasite that causes tropical theileriosis, a disease with worldwide economic impact in cattle. Tropical theileriosis is a vector-borne disease transmitted by Ixodid ticks and is highly prevalent in Africa, Southern Europe, the Near East, Far East and Central Asia including Iran. Theileriosis has considerable importance on livestock production due to high morbidity and mortality (1). *H. a. anatolicum* is considered one of the important tick vectors for *T. annulata* transmission in Iran and several countries (2–6).

There are three stages in *T. annulata* life cycle, release of infective sporozoites during tick feeding, sporozoite invasion to leukocytes and schizont developing and maturation of schizonts to merozoites for subsequent infection of red blood cells to form piroplasms (7).

Different control strategies for tropical theileriosis are developed including management, tick control, chemotherapy and immunization. Tick control is applied by use of acaricides but it is expensive and need to under supervised control measures and also is not sustainable. Clinical therapy is used for eliminating the clinical signs of disease by use of chemotherapeutic agents, but do not completely eradicate the *Theileria* parasites, and finally leading to the development of carrier state (8). Hence, vaccination is considered more cost-effective and environmentally safe strategy for disease control by administration of attenuated schizont-infected cell lines (9). The protective immunity can be demonstrated by challenge test through inoculation of highly virulent infective sporozoites to immunized animals (1).

However, challenge test by induction of experimental *Theileria* infection in calves may be achieved by the subcutaneous injection of ground-up infected tick stabilates or by the *T. annulata* infected blood samples (10). Nonetheless, the infective *T. annulata* sporozoites are accepted as a very virulent infective form of the parasite as a cryopreserved tick stabilate for challenge test. The infective sporozoites are usually produced by grinding the *Theileria* infected tick vectors (10, 11).

Bovine theileriosis vaccine has been manufacturing in Razi Vaccine and Serum Research Institute (RVSRI) since 1973, so the efficacy assay of the produced vaccine requires appropriate quality control tests including using virulent *T. annulata* strain for challenge test (1). Thus, tick-derived sporozoite in prepared stabilates should be produced in suitable quality and quantity for future plans such as evaluating the efficacy of live attenuated schizont infected cell line vaccine and evaluation of the efficacy of new proposed vaccine candidates.

The aim of this study was to determine the effectiveness of prepared cryopreserved tick infected stabilates by using molecular and cell culture as in vitro assays and injecting to naïve calves as an in vivo technique.

## Materials and Methods

This project was approved by ethical committee of Razi Vaccine and Serum Research Institute, No. RVSRI.REC.98.011 date 4 Nov. 2019.

### Preparation of ground-up tick stabilate

Highly virulent *T. annulata* infected blood samples have been kept in cell bank unit of the Parasite Vaccine Research and Production Department of Razi Vaccine and Serum Research Institute (RVSRI), Karaj, Iran. The virulent *T. annulata* Ak-93 strain has been isolated from an infected calf with severe theileriosis from Takistan, Qazvin Province, Iran in 2014. The infectivity and virulence of the Ak-93 strain were previously verified by in vivo calf inoculation.

An isolate of *H. a. anatolicum* tick was collected from a sheep in the village of Halajerd near Karaj, Alborz Province, Iran, during spring of 2017. The isolated tick was identified using the published taxonomic keys (12, 13).

Then, the laboratory rearing was started. Briefly, the engorged female collected tick was incubated at 28 °C and 80% relative humidity. The female tick began to lay a large amount of several thousand eggs. The eggs were hatched into six-legged larvae and the larvae have been fed on white rabbits to be engorged.

Rabbits were housed in a temperature and light-controlled animal room, under veterinary supervision. *Oryctalagus cuniculus*, Dutch rabbits aged 6 months and weighing between 2 and 2.5 kg, male were prepared from Razi vaccine and serum research institute, Department of Animal Husbandry and Nutrition.

Then, the larvae were taken and transferred to incubator. They molted to the nymphal stage. These hungry nymphs were kept to be infected by feeding on experimental *T. annulata* infected calf at the next stage.

A Friesian susceptible calf was used for induction of experimental theileriosis. A three month calf was provided from animal husbandry unit in Razi Vaccine and Serum Research Institute as a naïve healthy animal. The calf was monitored clinically and microscopically for *T. annulata* infection as well as specific PCR test for following tissue and blood protozoa in peripheral blood samples for one week before the test.

All of the procedures were performed in accordance with Animal Care and Ethics Committee (ACEC) of Razi vaccine and serum research institute guidelines. There was not any unnecessary manipulation or poorly designed animal experiments. Moreover, there was no deviation of designed protocols and working procedure and no animal wasting in research was happened. At the end of the experiments, all rabbits and calves were terminated according to the standard protocols.

The ground-up tick stabilate were prepared and cryopreserved in Razi Institute Karaj, Iran. Briefly, 20 ml *T. annulata-*infected blood was injected subcutaneously next to the prescapular lymph node of the susceptible calf. The body temperature was measured daily, after the rising of the body temperature; blood smears were prepared and stained with Giemsa solution. Then the smears were examined for *Theileria* parasite. The reared hungry nymphs were placed in an ear bag attached to the calf ear. After adequate feeding, engorged nymphs were brought to the incubator for molting. Then the adult ticks were applied to each ear of rabbit for sporozoites maturation. The ticks were washed and sterilized and then were ground up with cold RPMI media, centrifuged at 100 g for 5 min, and the supernatant was taken and passed through a 5μm filter. The provided suspensions were subjected to cryopreservation with the addition of 10% glycerol and 40% fetal bovine serum (FBS) and penicillin 100 IU/ml and streptomycin 100 ug/ml (1).

### To assess infectivity of cryopreserved tick stabilate

**In vivo assay:** After three weeks of cryopreservation of tick stabilates, a four months old Friesian healthy calf was injected for bioassay. The calf was kept under tick-free conditions and was free from *Theileria* infestation. A cryotube of prepared tick ground up stabilate (equivalent of 4 ticks/ ml) was thawed at room temperature and then was inoculated subcutaneously close to the prescapular lymph node. The rectal temperature was measured daily, and the temperature over 39.5 ºC was considered to be a fever. Upon rising temperature, the peripheral blood smears were collected from ears or tail for microscopic examination and molecular assay.

### In vitro assay

**Giemsa staining:** The biopsy materials from the swelling of lymph node were examined for the presence of schizonts of *Theileria*. The prepared blood smears were observed microscopically for erythrocytic piroplasms. The procedure of staining is performed briefly by adding Giemsa’s stain solution to methanol fixed tissue and blood smear slides and left for 20 min. Then, the slides were examined with magnification of 1000x.

**Methyl Green-Pyronin staining:** Preparing the salivary glands from the tick was performed by method of Edwards et al. Briefly, first, the heat melted paraffin is poured into the pyrex petri dish and allowed it to cool. The paraffin melted using a hot spatula for tick fixation. A tick was embedded in the melted paraffin by forceps. The scutum was removed with a microscalpel and the salivary glands were dissected and were placed into the phosphate-buffered saline (PBS) solution (14). The salivary glands were mounted on a microscope slide and were stained according to the method of Irvin et al. Then, the slides were air-dried and fixed for 2–5 min in Carnoy’s fixative solution, and were immersed for 2 min in 70% ethanol, and were immersed with distilled water for 2 min. The slides were immersed in 2% methyl green pyronine solution for 7–9 min and were rinsed in distilled water and air-dried (MGP Sigma-Aldrich, Germany) (15).

**PBMCs isolation:** The heparinized whole blood sample has been collected from the jugular vein of a naïve healthy Friesian calf and the peripheral blood mononuclear cells (PBMCs) were isolated by Ficoll/Paque solution and cryopreserved frozen (16).

**In vitro leukocyte transfection:** The number of 3×10^6^ PBMCs/ml were resuspended in RPMI-1640 medium supplemented 10% heat-inactivated fetal calf serum (FCS), and mixed with one cryotube of stabilate (equivalent to 4 ticks /ml) into the chambered cell culture slide. The mixture was incubated at humidified 37 °C incubator containing 5% CO_2_. Daily microscopic observation was performed to control the number, form and condition of the incubated cells. Mononuclear cell transformation by the infective tick stabilate in cell culture slide was monitored daily by an inverted microscope (17).

**RT-PCR assay:** Total RNA was extracted from the tick stabilate using the One Step RNA reagent (Bio Basic Inc. Canada) in accordance with the manufacturer’s instruction. DNase treatment was done to assure the RNA response in RT-PCR assay (DNase I, Jena Bioscience, Germany). The cDNA synthesis and further PCR were performed using the MMuLV reverse transcriptase and PCR mix (YTA, Iran) according to the manufacturer’s instructions.

The SPAG1 gene was used for detection of active *Theileria* infection (mRNA) in tick stabilate by using RT-PCR assay, but the cytochrome b and surface protein genes were used for verification of *Theileria* infection (DNA) in calf blood and tick samples by PCR. The specific *T. annulata* Oligonucleotide sequences are shown in Table 1 (18).

**Table 1:** The specific oligonucleotide primers were used for amplification of target cDNA and DNA in this study

***Primer***	***Sequence (5′- 3′)***	***Expected PCR product size (bp)***	***Reference***
SPAG-1 Forward	CTG GAC AAA TGG GTG AAG GAG	535	*T.annulata* sporozoite surface antigem (SPAG-1) M63017
SPAG-1 Reverse	GTC ATT TGT TGC GTA CTG TGC		
Tcytb Forward	ATG TGC CAG CAA AAG GTA TGG	990	*T.annulata* cytochrome b (Tcytb) M63015
Tcytb Reverse	AAA CTC CCC TCC ACT AAG CG		
SP Forward	AAT ACG CTC TAA CAT TGT TGG C	330	*T. annulata* surface protein precursor (TaSP) XM947650
SP Reverse	AAC AAC AAT CTT CGT TAA TGC G		

The PCR amplification was performed in the Gradient Palm-Cycler™ (Corbett life Science, Australia). The cycling conditions consisted of an initial denaturation at 94 °C for 3 min followed by 32 cycles of 95 °C for 10 sec, 55 °C for 30 sec, and 72 °C for 1 min and a final extension at 72 °C for 5 min for all three target gene sequences. The PCR products were electrophoresed on 1.5% agarose gel concentration and were stained through in-gel staining using Safe-Red^TM^ (CinnaGen, Iran) and visualized by UV Transilluminator (Uvidoc, Gel Documentation System, UK). The PCR products were sent for sequencing analysis to Bioneer Co., Korea.

### Nucleotide sequencing and phylogenetic analysis

The PCR product for local *T. annulata* cytochrome b gene was sequenced and was identified using the Basic Local Alignment Search Tool (BLAST) at the National Center for Biotechnology Information (NCBI). The phylogenetic analysis was performed based on *T. annulata* cytochrome b gene sequence using a maximum-likelihood approach (MEGA 6.0 software).

## Results

### The results of laboratory tick rearing and tick stabilate preparation

The procedure was carried out in different stages with partially overlapping. The clinical symptoms and parasitaemia were observed 10 d post-infection. This experimentally infected calf was subjected to a reliable source of virulent parasite for laboratory-reared *Hyalomma* ticks. The engorged female's ticks were successfully provided a batch of laboratory tick group. While the experimental theileriosis occurred, the laboratory-reared hungry nymph ticks were infected by blood-feeding. The engorged nymphs were collected and molted into adults. Finally, the hungry adult ticks were used for tick-stabilate, and were cryopreserved in liquid nitrogen.

**Fig. 1:**
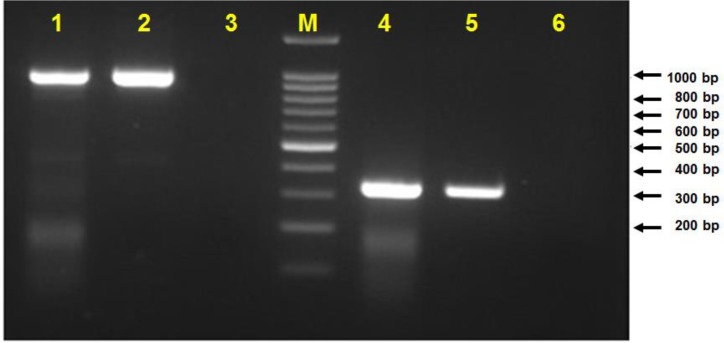
Agarose gel electrophoresis of PCR product of *T. annulata* infected tick DNAs. Lanes 1 and 2 are the positive results by using *T. annulata* cytochrome b primers and lane 3 is the negative control, lane M is the 100 bp DNA size marker (Thermo scientific, GeneRuler 100bp), and lanes 4 and 5 are the amplified tick DNA by *T. annulata* surface protein primers and lane 6 is the negative control

*Theileria* infection was confirmed by PCR genomic DNA assay by using cytochrome b and surface protein genes (Fig. 1). The cytochrome b gene sequence of the *T. annulata* Iran local strain has been submitted to Gen-Bank and can be retrieved under sequence ID of MH248139. The Iranian local isolate and several deposited *T. annulata* cytochrome b gene sequences from India, Iran, China, Tunisia, and Spain were used for this phylogenetic study.

### The results of bioassays for confirmation of tick stabilate infection

A prepared cryopreserved *Theileria* infected tick stabilate was inoculated to a naïve healthy calf by subcutaneous injection for *in vivo* bioassay for verification of tick stabilate infection to *T. annulata*. The results showed that the tick stabilate was capable to induce acute theileriosis. After a short period of time “thirteen days” post-injection the calf showed severe clinical symptoms of theileriosis.

**Fig. 2:**
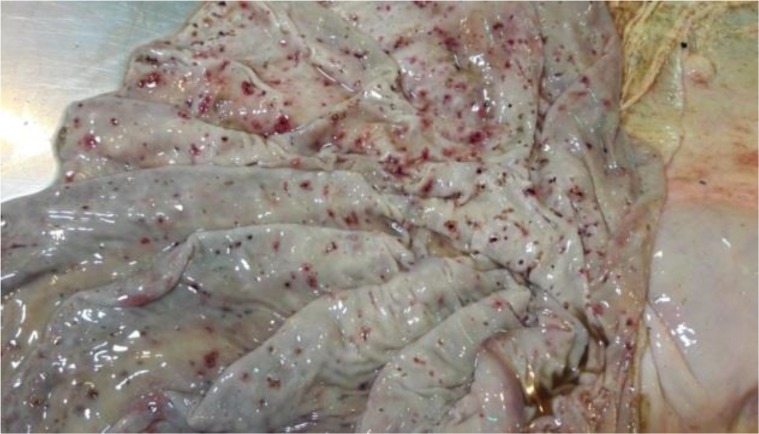
The abomasum of autopsied *T. annulata* infected calf that died due to severe theileriosis post tick stabilate injection. The numerous hemorrhage and ulcers are prominent on the mucous membrane of the inner layer (original)

The infection was confirmed by clinical and paraclinical including microscopic and molecular analysis. The most prominent clinical findings were fever, lymph node enlargement, inappetence, tachycardia, petechial and echymotic hemorrhagic foci on the mucous membranes, and icterus and finally death (Fig. 2). The autopsy was carried out immediately after death in pathology department of Razi institute and the vast dissemination of the parasite and hemorrhage have been shown in all the visceral organs including abomasum, intestines, mucous membranes, kidneys, liver, heart, and lungs (Fig. 2).

**Fig. 3:**
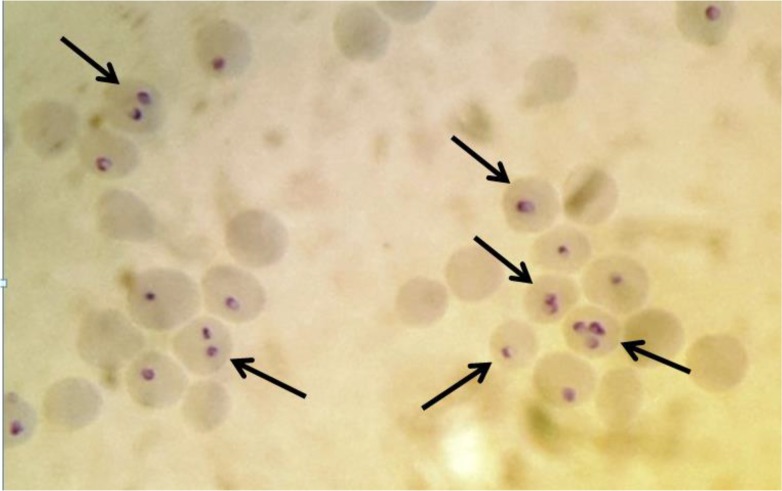
The Giemsa stained blood smear of *T. annulata* infected calf after 10 d of tick stabilate injection. The intraerythrocytic parasites (piroplasms) are shown by arrows; one, two and even three parasites are seen in infected red blood cells (original)

**Fig. 4:**
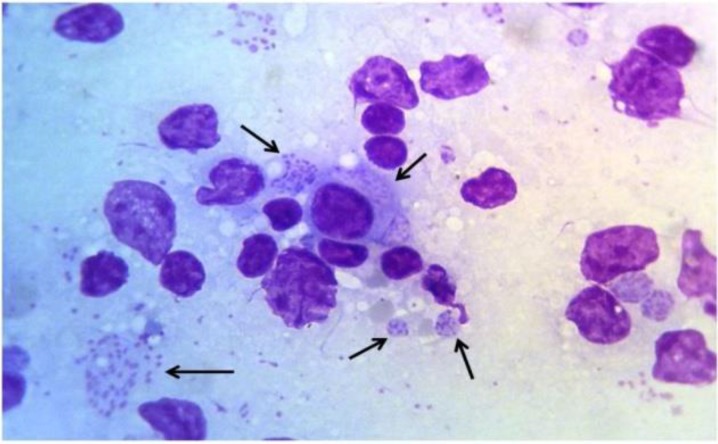
Giemsa stained lymph node smear of infected calf after 8 d post tick stabilate injection (original)

The *T. annulata* schizonts and piroplasms were detected microscopically in Giemsa stained blood smears, lymph node aspirated materials and visceral organs. The severe parasitaemia was shown by peripheral blood smears up to 40% of RBCs (Fig. 3 and 4).

**Fig. 5:**
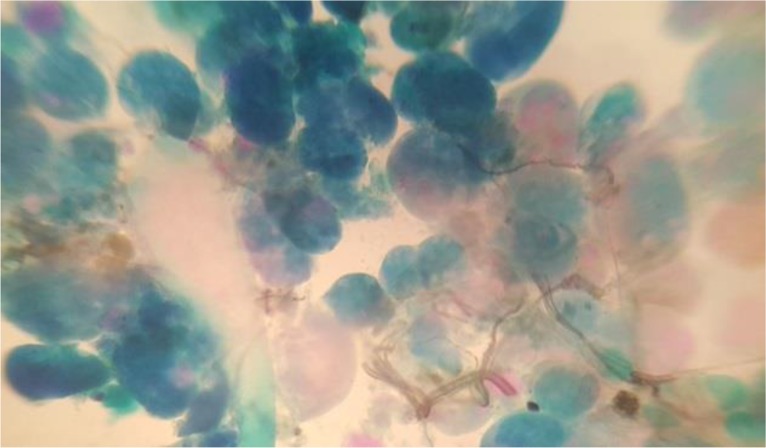
Methyl Green Pyronin stained salivary gland showing *Theileria* sporoblast infected acini harvested from *H. a. anatolicum* (original)

A number of engorged ticks were dissected for identification of salivary glands. The MGP stained salivary glands showed the presence of suspected infected acini by presenting the mass of *Theileria* sporoblast stained bluish green (Fig. 5).

### The results of in vitro tests for confirmation of tick stabilate infection

**Fig. 6:**
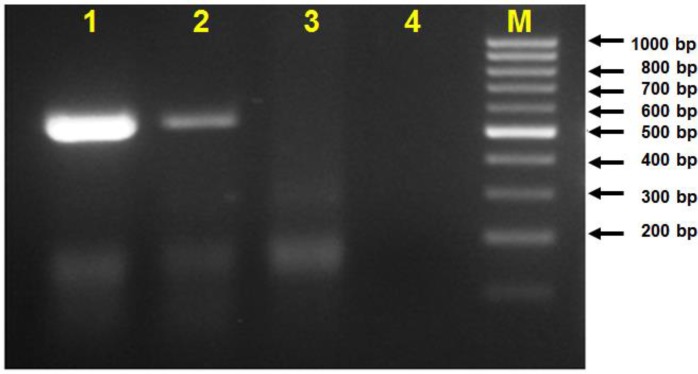
Gel agarose electrophoresis of RT-PCR assay for two produced tick stabilates in comparison with *T. annulata* infected tick DNA and negative control. Lanes 1 and 2 are the RT-PCR results for SPAG1 gene amplified for the *T. annulata* infected tick stabilates; lanes 3 is the results of RT-PCR for *T. annulata* infected tick DNA and lane 4 is the negative control (no template), and lane M is 100 bp DNA size marker (Thermo scientific, GeneRuler 100bp)

The RT-PCR assay was performed for demonstrating sporozoite RNA in prepared whole body tick stabilate as well as dissected salivary gland acini. The total RNA isolated and used for cDNA synthesis. The application of sporozoite antigen gene as a target gene in RT-PCR assay confirmed the presence of infective parasite in prepared tick stabilate (Fig. 6).

**Fig. 7:**
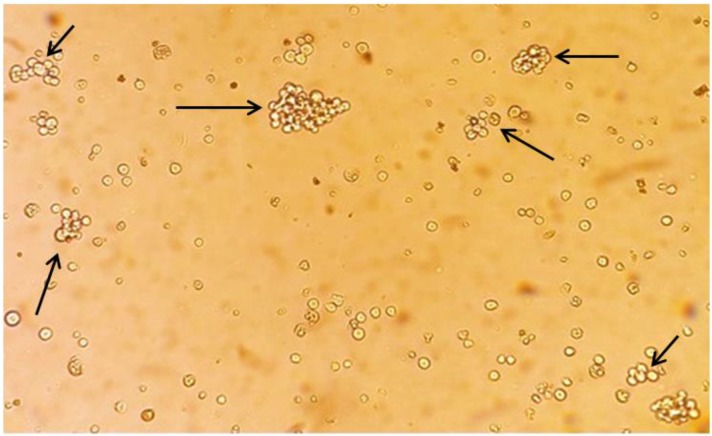
*T. annulata* schizont infected cell line “Halajerd”, passage number 1 in stoker medium. The transformed naïve bovine PBMCs by *T. annulata* live sporozoites via tick stabilate were proliferated are presented in clump form by arrows. The image was prepared four days post *in vitro* cell transfection (original)

*T. annulata* infected cell lines were achieved by in vitro infection of PBMCs with the prepared tick stabilate. The transformed cells were proliferated and formed clumps two days post-inoculation. These clumps were *T. annulata* infected transformed cells. The transformed *Theileria* infected cell lines were proliferated and the samples were taken for cryopreservation in different passage numbers for further analysis (Fig. 7).

The specific *Theileria* PCR on genomic DNA using *T. annulata* cytochrome b and surface protein genes and Giemsa staining were used for detection and identification of *T. annulata* infection in the produced cell line. Therefore, the cell staining and molecular assay have verified the presence of *Theileria* infection in produced cell line (Fig. 1, 3 and 4).

### The result of nucleotide sequencing and phylogenetic analysis

**Fig. 8:**
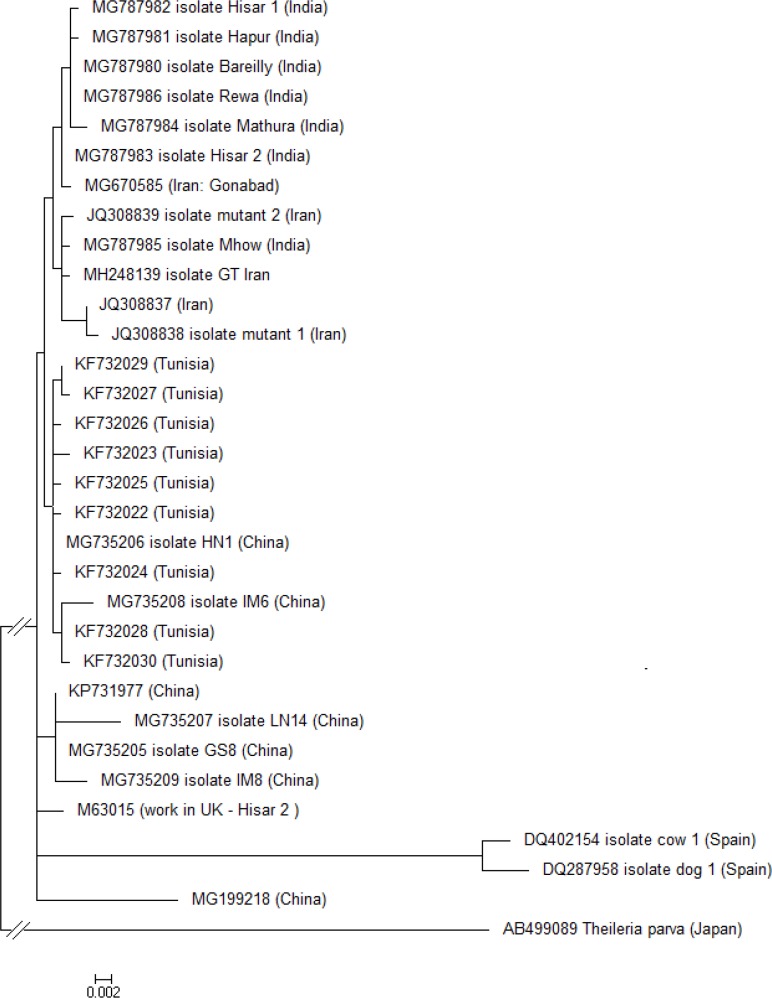
Rooted phylogenetic tree is constructed using *Theileria annulata* cytochrome b gene sequences. Phylogenetic tree was generated using a Neighbor-Joining approach (MEGA 7.0). Analysis was based on partial-length coding sequence of *T. annulata* mitochondrial Cytochrome b gene, investigated in this study or obtained from publicly available sequence datasets (GenBank). The evolutionary distances were computed using the p-distance and Tamura 3-parameter models (with same topology). The phylogenetic tree was rooted at the midpoint. Branches are drawn in proportion to the scale bar and bar represents 0.02 nt substitutions. According to the constructed tree *T. annulata* strains and isolates were divided in three major clades that Iranian isolates were in this branch close to the Indian isolates

The 914 bp nucleotide sequence of *T. annulata* cytochrome b gene were compared with a several related cytochrome b gene sequences in GenBank database. The analysis showed a high percentage of similarity (99%) to other published sequences for cytochrome b from India, Tunisia, Iran and China. Phylogenetic analysis was performed based on the partial-length coding sequence of *T. annulata* mitochondrial cytochrome b gene. The Phylogenetic tree was constructed based on a GTR+I+G model and was midpoint rooted. The isolate from current study was clustered in a subclade of Iranian and Indian *T. annulata* isolates (Fig. 8).

## Discussion

The most important points of interest in this study, which distinguishes it from other cases in Iran are as follow: here is the first time, *T. annulata* sporozoite infected tick stabilate was produced and used for challenge test. Up to now, *T. annulata* schizont infected cell lines were prepared via aspiration biopsy from diseased animals but here for the first time a new *T. annulata* infected cell line was prepared in laboratory by in vitro technique. The standardization of the procedure was validated here for all stages by appropriate updated tests and assays: molecular DNA and RNA verification tests, microscopic observation on Giemsa stained blood smears and tick gland acini MGP staining, in vivo assay for determination of infectivity of prepared tick stabilate, vaccine challenge of immunized calf, and postmortem analysis.

In this study, a local *T. annulata* infected tick stabilate was produced and was cryopreserved in liquid nitrogen (−196 ^o^C). The produced tick stabilate has been shown that efficiently infect naïve and healthy calf to acute tropical theileriosis. Moreover, for in vitro assay, the cryopreserved naïve PBMCs isolated from healthy calves have been shown to be infected and transformed by the prepared tick stabilate in laboratory conditions. Therefore, we established a procedure for production of a reliable collection of *T. annulata* infected tick stabilate for bovine tropical theileriosis vaccine efficacy assay and other research and development studies.

Our findings are in agreement with results of other studies where successfully prepared a stabilate from *H. a. anatolicum* ticks infected with *T. annulata* for the challenge test, contained between 2.5 and 25 ticks/ml (19, 20).

However, different *T. annulata*-infected ticks were used for stabilate preparation; Gill et al. were prepared stabilates from *H. dromedarii* tick infected with *T. annulata*. They observed that one-tick/ml stabilate produced mild infection, but infection by 10-ticks/ml stabilate gave rise to severe type of the disease (10), Pipano and Samish showed that *T. annulata* stabilate derived from infected *H. excavatum* ticks for cattle infection (21, 22)

In the present study, the acute bovine theileriosis was appeared after the tick stabilate inoculation equivalent to 4 ticks/ ml. The infected calf showed the severe typical symptoms of acute theileriosis and finally died 16 d post-infection.

In vitro assay offers a quick and easy alternative to animal testing. The in vitro assays give more accurate and cheaper than in vivo infections for tick stabilate efficacy testing. The in vitro assays provide more ethical and economical and they normally do not imply animal suffering or death. Nevertheless, in vitro assay does not give reliable evident of the immunogenic characteristics and the virulence of the stabilates. However, in vivo immunization trials will still remain necessary to provide the quality assurance for field immunization (23).

Calves inoculation with *Theileria*-infected tick stabilate may be replaced by the classical *Theileria*-infected blood or infecting living ticks on cattle. However, the advantages of the tick infected stabilate injection are: 1) the management of inoculated cattle is easier than those to require living ticks; 2) as the tick-infected stabilates were prepared in a batch cryopreserved numbers, the results might be reproducible and repeatable (11).

## Conclusion

In this research for the first time the sporoblast of the *T. annulata* local isolate was shown in *H. a. anatolicum* tick vector. The infectivity of prepared tick stabilate was confirmed by in vivo and in vitro methods as well as naïve PBMCs infection and transformation. These findings may be useful for vaccine efficacy tests and vaccine development studies.
